# Can Currently Available Non-invasive Continuous Blood Pressure Monitors Replace Invasive Measurement With an Arterial Catheter?

**DOI:** 10.7759/cureus.54707

**Published:** 2024-02-22

**Authors:** Payton Gore, Hong Liu, Christian Bohringer

**Affiliations:** 1 Anesthesiology, University of California Davis Medical Center, Sacramento, USA

**Keywords:** hemodynamic monitoring, blood pressure, non-invasive cardiac output, arterial line, continuous non-invasive blood pressure

## Abstract

Deviations from normal blood pressure (BP) during general anesthesia have been clearly linked to several adverse outcomes. Measuring BP accurately is therefore critically important for producing excellent outcomes in health care. Normal BP does not necessarily guarantee adequate organ perfusion however and adverse events have occurred even when BP seemed adequate. Invasive blood pressure monitoring has recently evolved beyond merely measuring BP. Arterial line-derived pulse contour analysis is used now to assess both cardiac output and stroke volume variation as indices of adequate intravascular volume. Confirmation of acceptable cardiac output with data derived from invasive intra-arterial catheters has become very important when managing high-risk patients. Newer devices that measure BP continuously and non-invasively in the digital arteries via a finger cuff have also become available. Many clinicians contemplate now if these new devices are ready to replace invasive monitoring with an arterial catheter.

Unlike non-invasive devices, intra-arterial catheters allow frequent blood sampling. This makes it possible to assess vital parameters like pH, hemoglobin concentration, ionized calcium, potassium, glucose, and arterial partial pressure of oxygen and carbon dioxide frequently. Non-invasive continuous BP measurement has been found to be unreliable in critically ill patients, the elderly, and patients with calcified arteries. Pulse contour-derived estimates of cardiac output and stroke volume variation have been validated better with data derived from arterial lines than that from the newer finger cuff monitors. Significant advances have been recently made with non-invasive continuous BP monitors. Invasive monitoring with an arterial line however remains the gold standard for measuring BP and assessing pulse contour analysis-derived hemodynamic variables in critically ill patients. In the future, non-invasive continuous BP monitors will likely replace intermittent oscillometers in the operating room and the postoperative period. They will however not eliminate the need for arterial catheterization in critically ill patients.

## Introduction and background

Deviations from normal blood pressure (BP) during general anesthesia have recently been linked to several adverse outcomes such as acute kidney injury (AKI), myocardial infarction (MI), postoperative cognitive dysfunction (POCD), stroke, and death [1-5]. Measuring BP accurately during and after anesthesia is therefore very important for providing safe care to patients who are at high risk of suffering these adverse outcomes. BP can be measured non-invasively by either intermittent or continuous cuff-based techniques. Alternatively, an arterial catheter can be inserted into an artery and the BP can be monitored directly via a transducer. This direct measurement via an arterial cannula is currently still regarded as the reference method when testing new devices (criterion standard) [6]. Invasive BP monitoring and its use have evolved over time and the pulse contour is now commonly analyzed to derive blood flow and dynamic indices of intravascular volume. This assessment uses proprietary computer algorithms to evaluate the area under the curve of the arterial pressure versus time graph. Measurement of cardiac output and stroke volume variation derived from pulse contour analysis is now actually recommended for perioperative fluid management in high-risk patients [7]. A number of non-invasive continuous BP monitors are currently available. Table 1 lists continuous non-invasive BP monitoring devices. Non-invasive continuous BP measurement has also been used to assess perfusion [8]. It has been validated in cardiothoracic surgery [9]. The emergence of these new continuous non-invasive techniques for measuring BP has led clinicians to wonder if these devices are sufficiently robust to replace intermittent cuff measurements in the majority or to avoid arterial cannulation in the (critically ill) minority of their patients. These new monitors may allow for better perioperative BP surveillance not just in high-risk patients but also in situations where an arterial line is not indicated. New non-invasive cuffless BP monitors are also under development [10]. This paper explains why an arterial line remains the monitor of choice in critically ill patients. This review is based on papers indexed in PubMed until December 2023.

**Table 1:** Currently available continuous non-invasive BP monitors This is a list of currently available continuous non-invasive BP monitoring devices and their measurement technology.

Monitor	Measurement technology
ClearSight (Edwards)	Volume clamping
CNAP (Biopac)	Volume clamping
Nano (ADInstruments)	Volume clamping
VitalStream	Pulse decomposition analysis

## Review

Limitations of non-invasive methods

*Accuracy of BP* *Measurement*

Intermittent automated oscillometric BP measurement with a cuff overestimates low BP and underestimates high BP when compared to invasive BP measurement [11,12]. In critically ill children, non-invasive oscillometric BP measurement was also found to be too inaccurate to guide management when compared to an optimally damped arterial line [13]. A significant discrepancy between oscillometric and intra-arterial pressure was also identified in a neonatal intensive care unit (ICU) [14]. Intra-arterial BP measurement is therefore recommended whenever BP values seriously influence the therapeutic management [14]. Increasing discrepancy between invasive and non-invasive oscillometric methods has also been found with increasing age [15]. When treating critically ill elderly patients, clinicians should therefore have a low threshold for placing an arterial line. In a recent meta-analysis, continuous non-invasive BP measurement with a finger cuff was also judged to be less accurate and precise than what was defined as acceptable at the beginning of the study. The bias and the standard deviation for the mean BP were 3.9 and 8.7 mmHg (95% limits of agreement -13.1 to 21 mmHg) [16]. Even though further refinements of continuous non-invasive monitors will be necessary, these devices can however successfully track the direction of BP changes in real time, and this is an undeniable asset [17].

Accuracy of Cardiac Output Measurement

**Figure 1:**
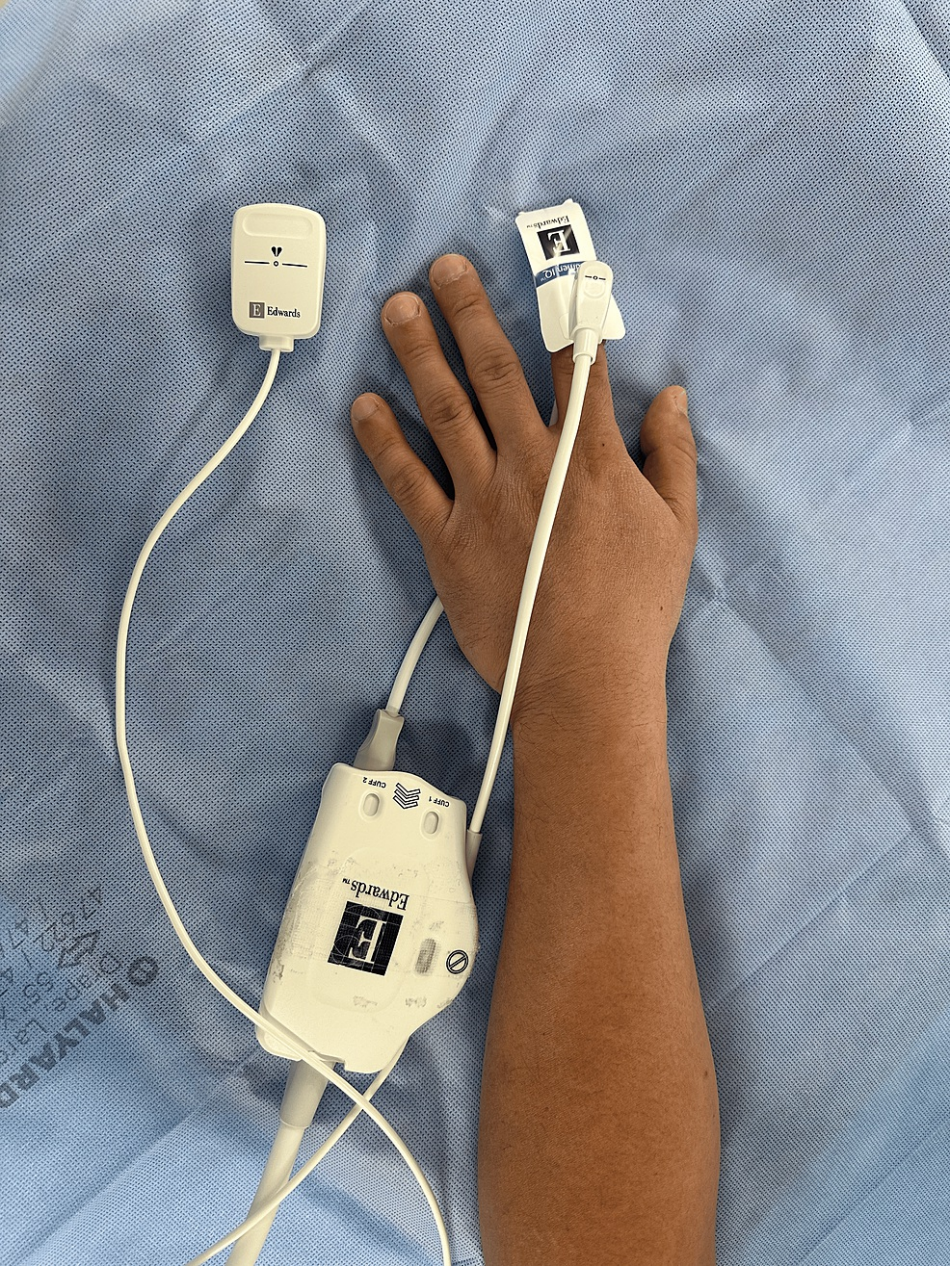
The ClearSight non-invasive continuous BP monitor The ClearSight non-invasive BP monitor uses the volume clamp technique and has a heart reference center (HRS) shown on the left that needs to be placed at the level of the heart.

Pulse contour-derived estimates of cardiac output and stroke volume variation have been validated better with data from arterial lines than that from the newer finger cuff monitors. Nevertheless, recent studies found reasonable agreement between non-invasive ClearSight finger cuff (Edwards LifeSciences LLC, CA)-measured cardiac output and bolus thermodilution measurements [18-20]. Finger cuff technology does expand the opportunity to monitor cardiac output and dynamic parameters of intravascular volume to a much larger group of patients because it does not require an arterial line or a pulmonary artery catheter. Further studies are needed however for clinicians to gain confidence in finger cuff-derived hemodynamic parameters. Figure 1 shows the ClearSight continuous non-invasive device.

*Direct Intra-arterial Measurement Is Necessary When Arteries Are Calcified* 

Calcium deposits in the arterial wall stiffen the blood vessel and increase the external pressure required to occlude the artery. A cuff-based oscillometer relies on external pressure to occlude the artery and this can produce erroneous results. In patients with radiological evidence of arterial calcification, indirectly measured BP via an upper arm cuff was found to overestimate intra-arterial BP for values under 150 mmHg and to underestimate BP above 150 mmHg. Invasive intra-arterial BP measurement is therefore necessary for obtaining accurate results in this group of patients [21]. In some patients, the cuff pressure cannot occlude the calcified artery completely. This not only makes it difficult to measure BP but may also prevent effective control of bleeding with an arterial tourniquet [22]. Finger cuff-based noninvasive methods use a volume clamp technique however and they therefore do not rely on occluding the artery under the cuff. Arterial calcification therefore theoretically does not pose a problem for this new technique, even though this issue remains to be investigated systematically. 

*Transducer Level * 

**Figure 2:**
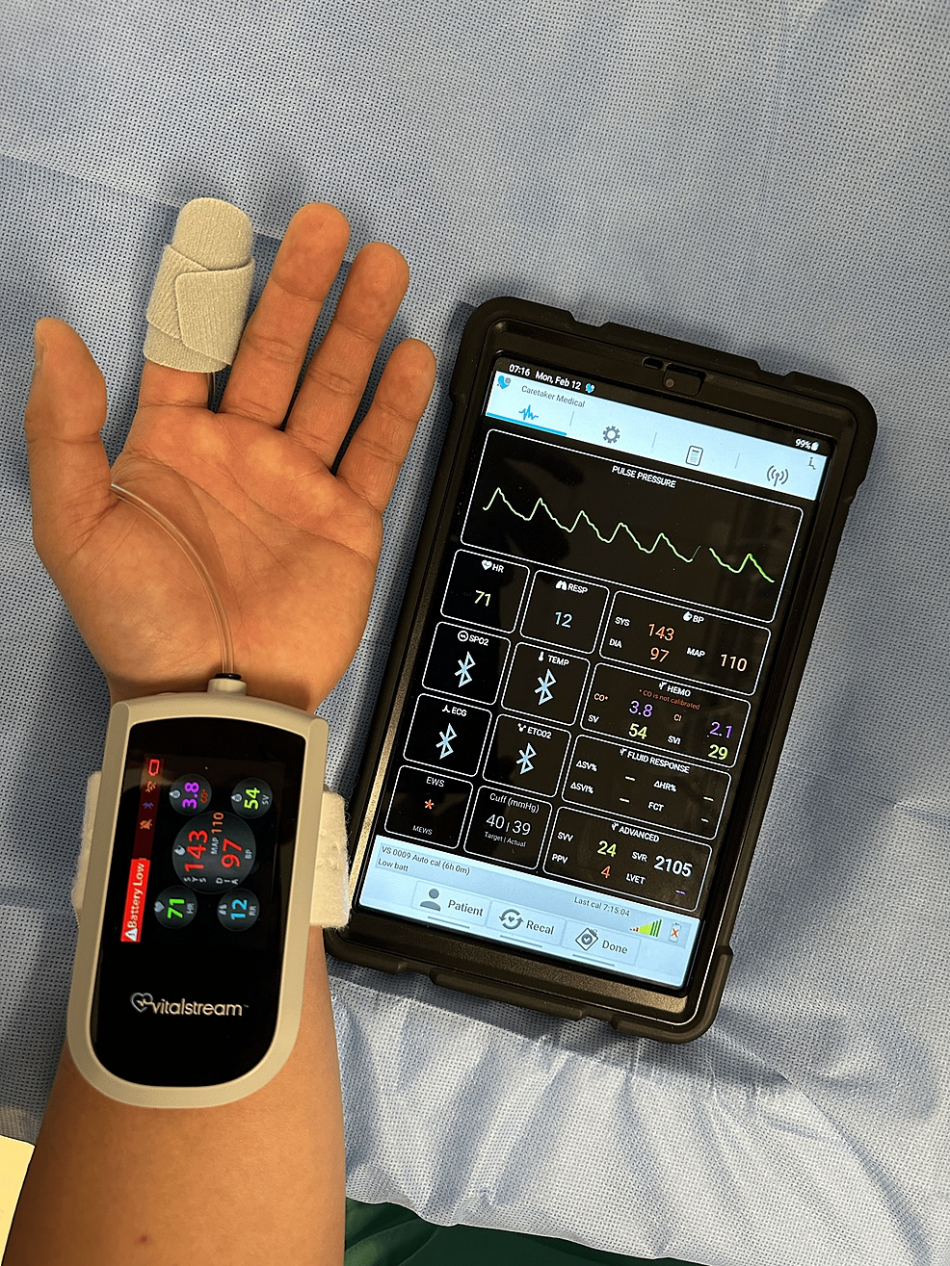
VitalStream continuous non-invasive BP monitor The VitalStream continuous non-invasive BP monitor uses pulse decomposition analysis technology and therefore does not need a heart reference center. It requires re-zeroing however if the patient is placed in the sitting position. This system is not tethered to a monitor by a cable and therefore is ideal for monitoring ambulatory patients. Data is transferred wirelessly to a tablet that is also shown in the picture and to an electronic medical record system.

An invasive arterial BP measurement system allows for adjusting the height of the transducer. This is an advantage when the patient is repositioned frequently to the sitting or the reverse Trendelenburg positions. Changing the position can create a significant hydrostatic pressure difference between the brain and the brachial or the finger cuff. When zeroing the transducer, the system needs to be open to air at atmospheric pressure. The transducer needs to be placed at the height level of interest during this process [23]. With the patient in the sitting position, the transducer is normally zeroed at the level of the external ear. The non-invasive ClearSight finger cuff also has a transducer that can be leveled to any height level of interest, such as the right heart, or if preferred the cerebral arteries [24,25]. VitalStream by Caretaker Medical (Charlottesville, VA) is a different continuous non-invasive finger cuff monitor that is based on the principle of pulse decomposition analysis and does not have a transducer that can be zeroed [26-28]. This system therefore should be re-calibrated after the patient has been placed in the sitting position. Figure 2 shows the VitalStream continuous non-invasive device.

Arterial Cannulation Allows for Serial Analysis of Blood Samples

Direct arterial catheterization greatly facilitates frequent blood sampling for measuring pH, lactate, hemoglobin concentration, ionized calcium, and arterial partial pressure of oxygen and carbon dioxide. Measuring these parameters is especially helpful in patients with low cardiac output that is insufficient for tissue oxygenation and metabolic requirements. The patient may be in circulatory shock even when BP appears to be acceptable. Patients with respiratory impairment also benefit greatly from serial blood gas analysis. Assessing serial blood gases allows quantifying the response to treatment and helps determine if the patient needs to be intubated or remain on positive pressure ventilation.

Arterial Lines Avoid Some Errors of BP Cuff Measurement

Invasive BP measurement also avoids problems arising from inappropriate upper arm cuff size. Cuffs that are too small overestimate BP and cuffs that are too large underestimate BP. The commonly available upper arm cuff sizes do not fit many patients [29]. The BP is often measured falsely high in obese patients because the cuff size is not adjusted for their arm circumference [30]. The century-old issue of how to select the right size arm cuff for accurate BP measurement remains unresolved [31]. The tubing of the BP cuff also frequently gets kinked when the arm is tucked, and this prevents the system from working. The cuff may need to be inverted so that the hose points directly toward the monitor. Surgeons or surgical assistants may inadvertently bump the cuff while it is cycling. Intermittent non-invasive BP systems rely on a constant signal from the arterial pulse, and they cannot cope with this type of interference. If the cuff was disturbed during the measurement cycle, a new reading must be obtained while carefully avoiding external interference with the cuff. Invasive and continuous non-invasive techniques are affected less by this issue because they provide a beat-to-beat measurement. Arrhythmia-induced beat-to-beat variability of the pulse wave also complicates measurement with intermittent oscillometers [32]. When pulse amplitude varies significantly from beat to beat, the monitor often does not provide a reading but gives an error message instead. The cuff then must cycle several times before a reading is finally obtained and this may delay detecting hypotension. A recent study in critical care patients found no difference in the accuracy and precision of readings taken by intermittent cuff technique in patients with arrhythmia and those with a regular rhythm [33]. Measurements obtained by oscillometers during dysrhythmia therefore can be regarded as reliable if the system delivers a reading. Continuous non-invasive monitors cope better with arrhythmias because they provide a beat-to-beat measurement.

Potential problems of invasive intra-arterial BP measurement

An analysis of the relative strength of invasive versus non-invasive methods of measuring BP warrants a discussion of the potential complications with invasive measurement.

Thrombosis 

Arteries may occlude after being cannulated with a catheter. This complication occurs more often in neonates and children than in adults [34]. Patients with scleroderma are also at increased risk of limb ischemia and arterial cannulation should be avoided in these patients [35]. The radial or the dorsalis pedis artery is preferred for catheterization because collateral blood supply from the ulnar or the posterior tibial artery reduces the risk of ischemic complications. Perfusion of the hand or the foot usually remains intact even when the radial or the dorsalis pedis artery is completely occluded. Performing an Allen test prior to insertion to confirm adequate collateral circulation has been found to be operator-dependent and unreliable as a means of predicting ischemic complications [36,37]. An intact pulse oximeter plethysmography trace from a probe applied to an ipsilateral finger after compression of the radial artery may be a more reliable indicator of adequate collateral flow.

Intra-arterial Injection

Staff need to be very vigilant to prevent accidental inadvertent intra-arterial injection of air bubbles or medications intended for intravenous administration. Both can lead to thromboembolic complications. The thrombosis frequently results in significant ischemia that requires part of the extremity to be amputated [38-41].

Inadvertent Disconnection

Inadvertent disconnection of the arterial line tubing causes rapid blood loss. All Luer lock connections need to be tightened thoroughly to prevent this. The cap on the three-way stop cock should not be perforated. Some kits come with a perforated cap on the three-way stop cock so that the cap does not need to be removed when zeroing the system. A perforated cap exposes the patient to the risk of sudden major hemorrhage however if the stop cock is inadvertently turned to the open position in the postoperative period. This can happen easily when the patient is agitated, and the stop cock is taped to the patient’s forearm.

Failure to Cannulate an Artery

Occasionally it is very difficult or even impossible to cannulate an artery. The risk-benefit ratio of an arterial line needs to be assessed carefully for every individual patient. If the patient is shocked and radial and dorsalis pedis attempts have failed, then the catheter should be placed into the femoral artery despite the increased risk of hemorrhage and infection in this location. If frequent blood samplings are not necessary, a non-invasive continuous monitor can be used instead. Clinicians now frequently use non-invasive finger cuff devices for continuous BP monitoring when they are unable to place an arterial line.

Infection

When arterial lines remain in situ for several days, there is a risk of infection with *Staphylococcus aureus*. This hospital-acquired infection is associated with significant morbidity, mortality, and cost. Arterial lines are an underappreciated source of bloodstream infections [42]. Care needs to be taken to insert arterial catheters under sterile conditions. They also need to be assessed regularly for signs of developing infection.

Resonance and Damping Phenomena

Invasive BP measurement is very prone to damping and hyperresonance artifacts. The morphology of the BP trace therefore needs to be carefully assessed when making treatment decisions based on the systolic rather than the mean BP [43]. Non-invasive methods of continuous BP measurement are less prone to hyperresonance, and this is an advantage when staff do not understand the principles of wave summation. 

Contraindications

When the radial artery has been harvested as a conduit for a coronary artery bypass graft, this site can no longer be accessed for invasive BP monitoring. The ipsilateral ulnar artery enlarges to compensate for the absence of the radial artery [36]. The ipsilateral ulnar artery also should not be cannulated when the radial artery has been harvested as a conduit or has been tied off after a traumatic injury. Patients with scleroderma and Raynaud’s phenomenon are also at increased risk of ischemic complications and arterial cannulation should be avoided in these patients [35].

Table 2 compares the advantages and disadvantages of continuous non-invasive BP monitors.

**Table 2:** Advantages and disadvantages of continuous non-invasive BP monitors This table summarizes the advantages and disadvantages of continuous non-invasive BP measurement devices.

Advantages	Disadvantages
Measures BP continuously unlike oscillometer	Does not allow blood sampling
Easier to find a cuff that fits a finger than the arm in obese patients	Less accurate than invasive BP in the critically ill
Allows continuous measurement when arterial cannulation has failed	Unreliable with calcified arteries
No infection, bleeding, or thrombotic complications as seen with an arterial line	Less accurate in the elderly
Less prone to wave summation artifacts than an arterial line	Pulse contour-derived cardiac output better validated with an arterial line

## Conclusions

Measuring BP with an arterial line is still the gold standard despite the significant advances made recently by non-invasive continuous BP monitoring technologies. Unlike an arterial line, the non-invasive continuous monitors do not allow for frequent blood sampling. Serial blood gas analysis is often necessary however when treating high-risk patients. The new non-invasive continuous BP monitors therefore will not replace arterial catheters in critically ill patients in the ICU or the operating room. They do however offer significant advantages over standard oscillometers in healthy patients not suffering from massive hemorrhage, respiratory failure, or shock. These new continuous non-invasive monitors are therefore likely to replace intermittent measurement with a BP cuff in the future not only in the operating room but also during the entire postoperative period. Unlike oscillometers, continuous finger cuff monitors do not cause patient discomfort while measuring BP and they are therefore very useful during monitored anesthesia care and when frequent measurements are necessary. Monitoring BP continuously expedites diagnosing both hypotension and hypertension. These new monitors can also assess blood flow, and this improves hemodynamic surveillance in patients who do not have an arterial line or a pulmonary artery catheter.
